# Autonomous robotic additive manufacturing through distributed model‐free deep reinforcement learning in computational design environments

**DOI:** 10.1007/s41693-022-00069-0

**Published:** 2022-05-23

**Authors:** Benjamin Felbrich, Tim Schork, Achim Menges

**Affiliations:** 1 University of Stuttgart, Institute for Computational Design and Construction, Stuttgart, Germany; 2 Cluster of Excellence IntCDC Integrative Computational Design and Construction for Architecture, University of Stuttgart, Stuttgart, Germany; 3 School of Architecture, Faculty of Design, Architecture and Building, University of Technology Sydney, Sydney, Australia

**Keywords:** Additive manufacturing, Robotic construction, Deep reinforcement learning, Distributed control, Computer-aided manufacturing

## Abstract

The objective of autonomous robotic additive manufacturing for construction in the architectural scale is currently being investigated in parts both within the research communities of computational design and robotic fabrication (CDRF) and deep reinforcement learning (DRL) in robotics. The presented study summarizes the relevant state of the art in both research areas and lays out how their respective accomplishments can be combined to achieve higher degrees of autonomy in robotic construction within the Architecture, Engineering and Construction (AEC) industry. A distributed control and communication infrastructure for agent training and task execution is presented, that leverages the potentials of combining tools, standards and algorithms of both fields. It is geared towards industrial CDRF applications. Using this framework, a robotic agent is trained to autonomously plan and build structures using two model-free DRL algorithms (TD3, SAC) in two case studies: robotic block stacking and sensor-adaptive 3D printing. The first case study serves to demonstrate the general applicability of computational design environments for DRL training and the comparative learning success of the utilized algorithms. Case study two highlights the benefit of our setup in terms of tool path planning, geometric state reconstruction, the incorporation of fabrication constraints and action evaluation as part of the training and execution process through parametric modeling routines. The study benefits from highly efficient geometry compression based on convolutional autoencoders (CAE) and signed distance fields (SDF), real-time physics simulation in CAD, industry-grade hardware control and distinct action complementation through geometric scripting. Most of the developed code is provided open source.

**Supplementary Information:**

The online version contains supplementary material available at 10.1007/s41693-022-00069-0.

## Introduction and context

### Planning and autonomy in computational design and robotic fabrication

With the rise of CDRF in architecture as an established research field in the last two decades, a fundamental shift of the AEC industry towards higher degrees of automation becomes apparent (Menges 2015; Willmann et al. 2014). Throughout the years, a higher degree of robotic autonomy was a persistent goal among researchers as it bears the potential to adapt construction logics to new robotic means of making and ultimately free up construction workers from repetitive and dangerous tasks. Numerous CDRF research projects demonstrated the use of robots for prefabrication and in situ construction with a wealth of different material systems and construction methods. General purpose industrial robot arms enjoy great popularity within this field of research as they possess high payloads, versatility and precision. The variety of investigated materials and structures reaches from experiments with robotic brick laying (Bonwetsch et al. 2007), composite fiber winding (Doerstelmann et al. 2015), metal welding (Parascho et al. 2018), timber sewing (Alvarez et al. 2019; Schwinn et al. 2016) to 3D printing of thermoplastics (Yuan et al. 2016), concrete (Khoshnevis et al. 2006) or composites (Felbrich et al. 2018b; Hack and Lauer 2014) and many more. These projects demonstrated the ability of the CDRF research community to conceptualize, develop and fully implement complex robotic fabrication processes. Often the machining capabilities of industrial robots are heavily extended through elaborate custom end effectors and sensor systems. However, robotic tool paths are often carefully crafted by a human adhering to a static unidirectional information flow from the CAD model of a desired product to machine instructions. This mode of operation has its roots in the adoption of numerical control (NC) in manufacturing since the 1940s and the subsequent development of CAD/CAM routines that offer a more streamlined way of instantiating virtual objects in the physical world using machines. As is the case in many manufacturing fields that involve heavy machinery, these workflows justifiably favor robustness, precision and security over adaptiveness and agility, which are desirable qualities in robotics research.

An increased popularity of machine learning (ML) tools in this field proves that CDRF researchers are well aware of the potentials that novel ML methods have for design optimization. Noteworthy examples include the detection of fabrication-relevant material features such as wood knots (Nagy 2017; Norlander et al. 2015) or masonry cracks (Chaiyasarn et al. 2018), the prediction of material behavior like metal rod bending (Smigielska 2018), the use of various neural networks for conceptual design generation (As et al. 2018; Mahankali et al. 2018) or evaluation (Tarabishy et al. 2020), or as a direct modeling aid through a brain-computer interface (Cutellic 2019) or augmented reality headsets (Felbrich et al. 2018a).

Projects with a stronger focus on ML-enabled robotics include Brugnaro and Hanna (2017), where researchers made use of a Deep Neural Network (DNN) to cross-map fabrication parameters in robotic wood chiseling. Harichandran et al. (2019) enhanced an exemplary task of lifting a scaffold structure with four distinct lifting machines using state vector machines (SVM). Rossi and Nicholas (2018) demonstrated a way to map the paths taken by a metal sheet robotically manoeuvered through an English Wheel to its deformation behavior caused by the exerted pressure. This gave insight into how complex, hard-to-simulate metal deformation under rolling/pressure relates to its final curvature. Tamke et al. (2018) show two distinct case studies. In the first project, DNNs are used within a computational form-finding process of a wall structure composed of bending-active tensile rod modules. The optimization procedure of the global structure was simplified and heavily sped up by classifying load cases on the module-level. The second project made use of DNNs to predict unfavorable spring-back of metal sheets after being deformed in a process of robotic incremental sheet forming (RISF).

Although the use of classic supervised learning with DNNs shown in these projects bears some potential for optimized planning, it (a) disregards modern machine learning research especially within the field of robotic learning and (b) leaves large potentials of higher robotic autonomy in fabrication untapped. Achieving higher degrees of robotic autonomy within CDRF is extremely hard, if not impossible, with currently used ML tools.

In the realm of additive manufacturing both Mozaffar et al. (2020) and Nicholas et al. (2020) exemplify a tendency of approaching individual aspects of the design-to-fabrication workflow in isolation by focusing on tool path optimization using DNNs and/or DRL. Jin et al. (2020) propose ML-based optimization strategies to solve three separate steps of the entire workflow: improving geometrical design, process parameter configuration, and in situ anomaly detection. A coherent overview of ML-related additive manufacturing research given in Goh et al. (2021) similarly divides the reviewed projects into design optimization, process optimization and in situ quality control. As such, projects often heavily focus on solving isolated manufacturing related engineering problems. They do not target a constructive robotic problem-solving strategy as it is the goal in DRL robotics research.

### Deep reinforcement learning in robotics

Supervised learning approaches with the mere use of non-linear function approximators like DNNs for classification—as it is current practice in CDRF—is not feasible in robotic construction: to control a robot with an encoded state-action mapping—i.e., a decision making strategy—in a DNN, one would have to generate a very large data set of state-action pairs for training. With increased data dimensionality and continuous action spaces, as they are common in CDRF, this approach of basically presenting the entire cause-reaction space to a DNN quickly becomes unfeasible. Furthermore, in many cases, a human instructor might not even have the insight to provide suitable actions for every possible environmental circumstance and rather wants the learning mechanism to find solutions to construction tasks by itself.

In this regard, reinforcement learning (RL)—first comprehensively described in Sutton and Barto (1998)—is a particularly promising subset of machine learning. A few key achievements of this approach are fully autonomous aerobatic flight control of a helicopter (Abbeel et al. 2007), robotic control through demonstration and imitation (Kober and Peters 2011), bipedal (Mordatch et al. 2016) and quadrupedal robotic locomotion (Haarnoja et al. 2018a; Hwangbo et al. 2019), the defeat of human players in many Atari games (Mnih et al. 2013) and even mastering the highly complex strategic board game Go on a super-human level—AlphaGo (Silver et al. 2017).

RL and Deep RL (DRL) can overcome the aforementioned limitations as they encourage an agent to independently explore the state space. As the robotic agent receives positive rewards for favorable actions and autonomously maximizes this accumulative reward through strategies such as state(-action) value estimation, policy gradients and/or model examination, it is the human’s only responsibility to lay out an appropriate environment and reward-granting logic, which can be tailored towards constructive tasks. The advantages of DRL to (a) operate in vast environments with continuous or practically infinitely large discrete state-action spaces and (b) discovery of strategies beyond human demonstration render it a promising means for robotic autonomy in construction.

A thorough overview over DRL-based robotic control paradigms is given in Amarjyoti (2017). Interestingly, DRL researchers at times choose construction tasks to practice and benchmark learning algorithms. A few of them will be mentioned here. A more in depth discussion of some individual algorithms applied will be given in Sect. 2.2.

With the focus on noise resilience and affordability, Deisenroth et al. (2011) presented a very sample-efficient way of training a noisy, low-cost gripper-equipped manipulator to build a tower of colored blocks through visual feedback. The custom-made framework used for this study, PILCO, was introduced earlier in a more general fashion (Deisenroth and Rasmussen 2011).

Duan et al. (2017) and Finn et al. (2017) presented a different approach of meta-learning in which an agent not only learns a policy but also a way to generalize said policy to new tasks. Here, a robot is able to learn the task of stacking blocks through a single demonstration by a human, giving rise to its name *one-shot imitation learning*. It uses two DNNs: a vision network to infer object positions from raw image data, and an imitation DNN to derive and generalize the task intent from the demonstration. The latter is pre-trained on thousands of simulated demonstration samples.

Liu et al. (2018) made use of Deep *Q*-Learning in which the prospective value of a state-action pair at the current time step (the *Q*-value) is predicted using a DNN. With this technique, the agent learns to plan the stacking of irregular 2D-objects into a stable wall. The trained planning policy is then executed by a robot arm.

Zhang et al. (2019) describe a method to learn state model representations from image data. Although the paper’s main focus is structured representation and inference of model dynamics, it also employs a construction task—connecting Lego bricks—as a benchmark (among others).

Since at least the introduction of one-shot imitation learning, the task of object stacking—the most fundamental form of additive construction—with full robotic autonomy and very little human guidance can be considered solved. As DRL researchers are interested in improving the performance of their algorithms, they naturally tend to employ these simpler construction tasks like block stacking and have little motivation to extend their research to other construction principles. It seems, however, that this research field bears enormous potentials in increasing robotic autonomy in CDRF. Still DRL found little resonance within the CDRF community so far.

### Motivation

The reasons why DRL has not been employed in CDRF yet are manifold. Both DRL and CDRF are relatively young research fields, with largely unrelated research focusses. Aside from the required educational background, the tools and methods that are used are quite different. A closer look at the latter is worthwhile.

In fact, both of these research areas heavily benefitted from recent developments of powerful software tools.

Aside from an increased availability of robotic hardware in CDRF, the rise of generative and parametric design software made it is easier than ever to ideate, shape and script architectural objects that are both highly optimized towards multiple objectives (structural performance, material efficiency, etc.) and at the same time robotically fabricable (Jabi et al. 2013; Braumann and Brell-Cokcan 2011). Especially, visual programming interfaces in CAD enjoy great popularity for their ease-of-use and modularity, with McNeel’s Rhino and Grasshopper (Rhino-GH) currently being the quasi-standard. Such tools enable designers to model, evaluate and improve design solutions through an extendible modular tool set that offers a multitude of relevant sub-functions such as simulation-based form finding, FEA, CAD/CAM, numerical optimization, daylight simulation, some basic machine learning and robot kinematics capabilities and others.

DRL research on the other hand heavily benefitted from the availability of optimized deep learning frameworks such as PyTorch, Tensorflow, Keras, Caffe and others. Their automatic differentiation capabilities make them highly modular as they allow for numerical optimization of arbitrarily composed neural architectures. In addition, the Robot Operation System ROS, the quasi-standard in experimental robotics, offers a multitude of middleware components to access and control practically every motor or sensor that is relevant in the field. It also provides very capable tools for robotic path planning, visualization, simulation and many more. These tools offer a much more agile framework than traditional NC that are common in CAD/CAM. Although their features are highly relevant in CDRF they are hardly used.

We thus conclude that in summation, all the relevant methodic as well as algorithmic foundations for robotic autonomy in construction are already around. They just exist in different research realms and have not been combined yet.

Buckminster Fuller, one of the most influential architects of the twentieth century said: “If you want to teach people a new way of thinking, don't bother trying to teach them. Instead, give them a tool, the use of which will lead to new ways of thinking.” (Senge et al. 1994, p. 28).

The direct contribution this study intends to make is a way of combining methods and tool sets of DRL and CDRF, that leverages their individual strengths. Our hope is that such a framework would allow practitioners and researchers outside the realm of DRL and robotic research to use state-of-the-art robot control and learning tools for robotic fabrication and CAD/CAM workflows. This bears the potential that (a) methods for multi-criterial (structural) performance analysis can be repurposed as training environments to highly efficient DRL optimization routines and robotic learning and thus lead to improved outcome and (b) serve as a means for DRL researchers to assemble training environments with direct practical relevance.

While the presented study aims to introduce a higher degree of autonomy into CDRF research, it considers practical requirements that are prevalent in typical industrial fabrication setups, such as the need for real-time control, geometric state representation, structural performance evaluation and tool path accuracy. Furthermore, it aims to provide insight into practical applications of DRL and the challenges therein.

For this purpose, a communication and control framework for distributed agent training and task execution is presented and demonstrated in two case studies.

### Related studies

As construction-related DRL research and ML-related CDRF research were already discussed, this section focuses on existing CDRF projects that made use of techniques and frameworks that are also common in DRL.

Robotic autonomy highly depends on sensor-based feedback-loops allowing an agent to react to unforeseen circumstances or reconsider its behavior. Thus, the integration of design and fabrication tools into continuous multi-directional workflows yields tremendous potentials for CDRF. Vasey et al. (2015) investigated the robotic placement of pre-impregnated polymer reinforced carbon fibers onto a pneumatic formwork. Using feedback from a load cell and adaptive control, robotic motion could be adjusted in reaction to formwork deformation or previous fiber displacement. Giulio Brugnaro et al. (2016) demonstrated adaptive robotic behaviors enabled by visual feedback in weaving bending rattan rods. In Heimig et al. (2020), images of an integrated camera were used to adapt tool paths in the highly complex task of 3d printing with metal.

A common challenge in sensor-based setups in CDRF is a multitude of available frameworks and products, all of which are not necessarily compatible. ROS was designed to overcome exactly these challenges by introducing a unified modular middleware that enjoys overwhelming support by hardware vendors and software developers. Both Feng et al. (2014) and Benjamin Felbrich et al. (2017) made use of ROS and ROS-supported sensor systems in setups related to architectural fabrication. The latter used ROS as the main communication infrastructure for a multi-machine fabrication setup involving two industrial robots, a custom-made UAV and tension sensing devices. The usefulness of ROS for human-aided fabrication with an augmented reality headset was successfully demonstrated in Wannemacher (2017) and Kyjanek et al. (2019). Sutjipto et al. (2019) demonstrated closed-loop, sensor-based 3D printing.

Gandia et al. (2019) implemented the powerful Open Motion Planning Library (OMPL) into a common CDRF workflow.

These studies allude to a process of general technical maturation through the incorporation of performant planning and control routines within robotic fabrication. The introduction of a unified platform implementing DRL learning and sophisticated control could help accelerate this process.

## Methods

### Distributed training-fabrication framework

The software infrastructure that was developed to facilitate the integrated training and fabrication process will be referred to as *deepbuilder*. It is presented in detail hereafter and demonstrated in video one.^1^

#### Design principles

To fully leverage the strengths of combining methods of both fields of research and reflect the needs of CDRF fabrication setups, our framework had to follow a few design principles, which then guided its specific layout:

*Access to algorithms*: Many newly developed DRL algorithms are provided to the community free and open source. The performance of these algorithms is often tested and demonstrated within the OpenAI Gym framework. It defines a simple interface of functions, a template for writing agent–environment interaction cycles. Adhering to this standard allows easy access to existing and newly developed algorithms in the future.

*Modular setup of training environments*: While powerful physics simulation frameworks such as MuJoCo, DART and ODE are commonly used among DRL researchers as environments for agent training, their capabilities for geometric modeling and performance analysis are limited. Rhino-GH on the other hand offers strong CDRF-related tools for modeling, analysis and simulation, an intuitive UI and is continuously extended by the community. The key idea is to turn Rhino-GH into a modular construction kit for training environments, i.e., an extension of OpenAI’s Gym concept into the realm of generative modeling and computational design. This opens up the possibility to assemble CDRF-related performance evaluation training fields for agent actions and also allows for the agent itself to perform script-based CAD modeling.

*Real-time physics*: While Rhino-GH offers great functionality, its physics simulation capabilities, especially in collision-rich scenarios do not match those of Gazebo or MuJoCo. However, robotic training scenarios and fabrication heavily rely on such assets. We thus extended Rhino-GH with a custom plugin for fast real-time physics simulation based on the Nvidia Flex engine (Benjamin Felbrich 2019).

*Open-endedness*: While our specific setup stipulated a certain CAD training environment, the framework should in principle be open to other means of simulation and action evaluation. Thus, language- and software-agnostic communication was to be favored wherever possible.

*Full integration with ROS*: ROS extinguishes itself through its modularity and community support within robotics. Making full use of its capabilities, especially in terms of motion planning and sensor control, greatly simplifies the execution of trained agent policies. For our experiments a Universal Robot UR10 with the appropriate ROS drivers was used. Further hardware choices will be discussed later.

*Support of real-time controls via fieldbus*: Although somewhat experimental, the presented research targets industry-grade machinery for its relevance in fabrication. The framework must, therefore, allow to automate a real-time fieldbus system such as EtherCAT.

*Bare-metal hardware support*: ROS-based hardware control requires a very stable network connection between the host and connected devices. Neural computation and physics simulation both heavily benefit from highly parallelized GPU computation. To fulfill these requirements, system virtualization through VMs or WSL was foreclosed in favor of dedicated hardware.

*Multiple simulation workers*: DRL algorithms, especially in model-free approaches, generally require a high amount of simulation steps, the reduction of necessary training samples and most efficient use of collected data is a major subject of investigation within DRL research. To compensate for the loss in simulation speed caused by network communication and system distribution, an ability to run multiple training sessions at once is crucial.

#### System overview

**Fig. 1:**
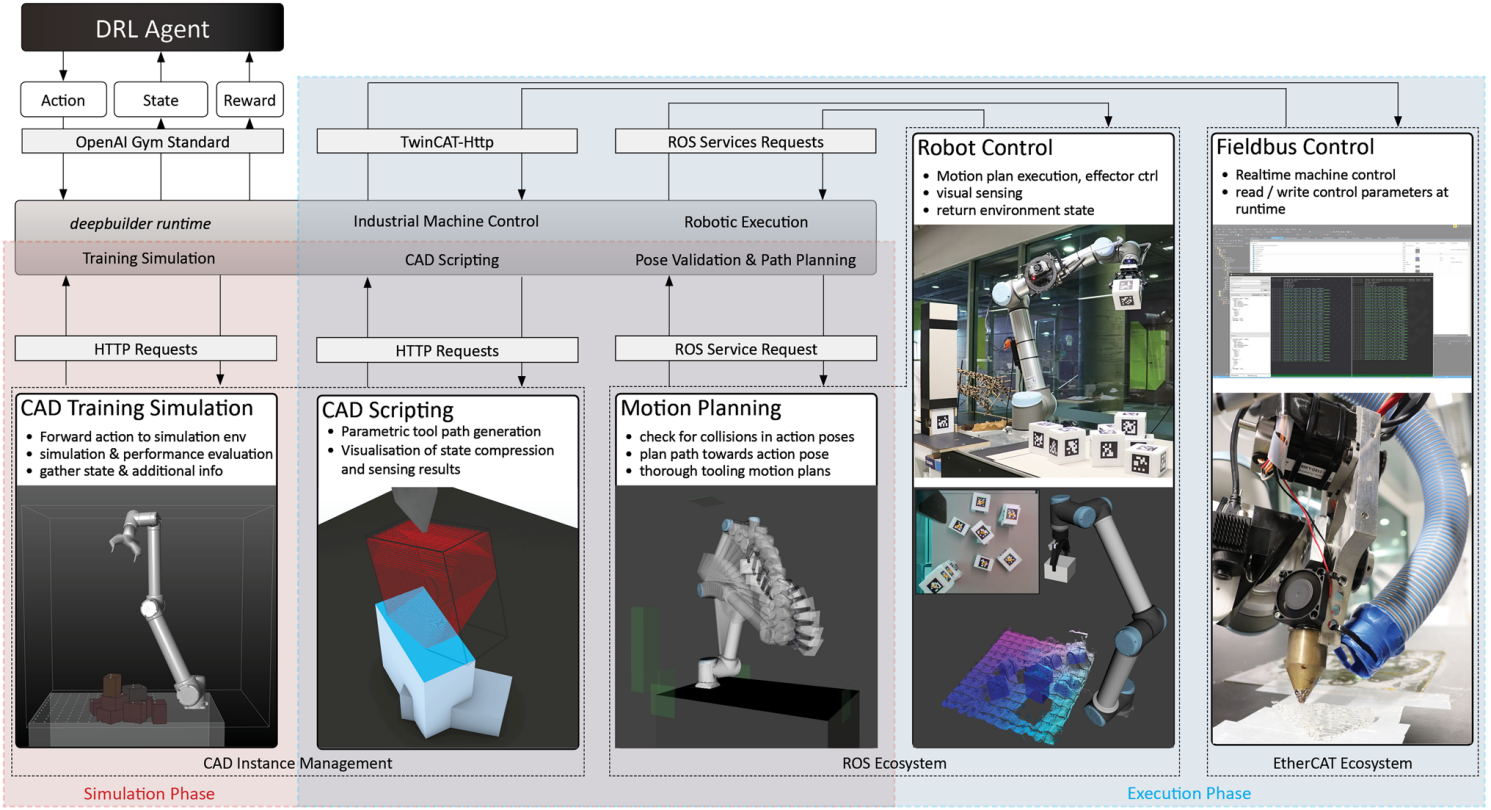
Deepbuilder system overview

With the discussed principles in mind, a system was established that can access and control a variety of relevant tools and applications (Fig. 1). This deepbuilder runtime partly consists of an environment class based on OpenAI Gym able to communicate with different tools and applications relevant to the task of robotic construction.^2^ It gives an autonomous DRL agent the ability to train its behavior within a simulation environment that offers parametric design features (i.e., a Rhino-GH script) while validating and planning movements through MoveIt, execute the trained policy on an actual robot and retrieve new environmental states through visual sensors. Furthermore, geometric CAD scripting routines can be automated to aid planning and execution, which is especially useful in critical tooling-related subroutines that require high precision. This enables the differentiation of building behavior into low-precision global movements, that are learned, and more precise local movements, that are scripted and complement the autonomous fabrication learning workflow. Lastly, a real-time middleware is connected to enable the control of industrial grade machinery.

*Simulation Phase*—A typical simulation phase incorporates the motion planning capabilities of OMPL in ROS to validate prospective actions in terms of collision avoidance with the environment through respective service calls. Actions that lead to collisions are considered bad and will be penalized. Collision-free actions are forwarded to the simulation environment through a CAD instance management service running on another computer in the network. There, Rhino-GH scripts can be used to (a) simulate the action, (b) infer performance-related information from the state geometry that is relevant for reward shaping and learning, and (c) derive refined tool paths in accordance with the environmental state if necessary.

*Execution Phase*—Once trained the policy is deployed to the actual hardware. Prior to execution each action is validated and planned through motion planning and possibly refined through CAD scripts. It is then executed by the robot arm and the respective ROS-driven hardware extensions. In cases where additional industrial hardware is needed throughout the action execution (e.g., controlling an extruder motor or heating a printing nozzle), respective commands are sent to another service allowing the control of fieldbus-connected devices.

#### System components

##### ROS setup^3^

For motion planning, a Bi-directional Transition-based Rapidly exploring Random Tree (BiTRRT) was used. When a desired goal could not be reached, a distinction is made between self-collisions, collisions with static environment objects (floor, virtual safety planes) and dynamic environment objects (objects added to the environment by agent actions). These differentiated measures can be used for nuanced reward shaping. ROS was used to control the UR10 robot, a gripper and sensing devices.

##### Sensor integration

For reliable camera-based pose estimation of physical objects, the popular visual fiducial system AprilTag (Wang and Olson 2016) was chosen and integrated into the system through the appropriate ROS drivers. Additional depth information gathered with the utilized Intel RealSense D435 camera was processed with custom-developed ROS nodes. They will be described in detail later.

##### CAD instance management

The simulation environment in CAD is accessed through an ASP.NET Web API running on a Windows computer. It serves three purposes: (1) instantiate and manage CAD instances making sure that there is an active application per training session running and ready to receive orders, (2) act as a microservice to receive Http requests from the deepbuilder runtime that include actions intended for simulation or scripting instructions intended to request further geometric information and forward them to an available CAD instance and (3) act as an instance watchdog, that detects faulty simulations, unresponsive Rhino-GH instances as well as excessive RAM usage and performs necessary supervisory measures such as killing and restarting simulation instances.^4^ Requests to the GH script were received through components implementing the .Net Http server functions.^5^ CAD instances are coupled to deepbuilder training sessions with unique identifiers. This allows for the parallel execution of multiple training sessions.

##### TwinCAT-Http-server^6^

EtherCAT fieldbus controllers can be programmed with Beckhoff’s TwinCAT3 over a real-time ethernet connection. It is a well-established industrial control tool. The related Beckhoff library TwinCAT.Ads offers the automation of TwinCAT through .Net languages such as C#. TwinCAT-Http-Server is a custom-developed WPF application that can communicate with TwinCAT.Ads while also acting as an ASP.NET Web API host receiving Http requests. It, therefore, exposes read and write functionality of TwinCAT3 parameters at runtime to arbitrary network locations in a language-agnostic way. In our case, the read and write requests were made from an accordingly designed ROS node and thus allowed ROS to remote control EtherCAT devices. TwinCAT-Http-Server also offers a UI to monitor traffic and plan correct requests.

### Choice of RL algorithms

Within the field of RL, many different algorithms have been proposed over the years. As it was not intended to introduce a new algorithm, the choice of a suitable existing one from the literature was crucial. A non-exhaustive overview of existing techniques is given in (Achiam 2018). An important distinction has to be made between model-based and model-free learning. In model-based RL, the agent retains an inner representation of the environment, a function that predicts state transitions to the next state $s{^{\prime}}$ and reward $r$ based on current state $s$ and action $a$. This enables planning and prediction of long-term strategies and thus yields a high sample efficiency. However, model-based approaches are generally more difficult to adapt to changing task definitions. As it was intended to test this learning framework on different construction tasks and possibly extend its use further, task-specific model implementation and tuning had to be avoided.

One model-free technique, *Q*-Learning, stores and incrementally improves the *Q*-function $Q(a,s)$ which approximates the value of state-action pairs, i.e., the total reward accumulation that can be expected after taking $a$ in state $s$ when following a given policy $\pi$. The optimal policy ${\pi }^{*}$ is the one that uses an optimal *Q*-function ${Q}^{*}$ to find the best possible action ${a}^{*}$ in every $s$. The main optimization objective is thus finding a *Q*-function that describes the task as complete and accurate as possible. Finding it is typically done by mediating between exploring the environment in early phases of training and later exploiting known information about advantageous actions. The classic form of *Q*-Learning, where *Q* values are stored in a table, is limited to discrete action spaces and thus not applicable to our task. However, using such state-action-value approximators, e.g., in the form of a DNN as its done in deep *Q*-Learning, is highly advantageous.

Policy optimization, also called policy gradient, techniques on the other hand, do not make use of such substitute optimization objectives and directly optimize the parameters $\theta$ that make up a policy to maximize accumulated reward. If the policy is represented by a DNN, $\theta$ are its weights.

For this study, we chose to use two modern model-free DRL algorithms that combine the advantages of policy optimization and value approximation: they are relatively simple to implement, yet provide state-of-the-art efficiency and do not require advanced task-specific model engineering: Twin Delayed Deep Deterministic Policy Gradient (TD3) (Fujimoto et al. 2018) and Soft Actor-Critic (SAC) (Haarnoja et al. 2018b).

As a successor to DDPG (Lillicrap et al. 2015)—a deep Q-Learning method adapted for continuous action spaces—,TD3 also concurrently trains neural approximators for ${Q}^{*}(s,a)$ and ${a}^{*}(s)$, but addresses DDPG’s high sensitivity to hyper-parameter tuning. It does so using two Q-DNNs, delaying policy updates and smoothing the target policy, to avoid Q-function error exploitation.

SAC is similarly structured, but differs in that it works with a stochastic policy, whose entropy is maximized along with reward accumulation throughout training. A maximized policy entropy is understood to be more expressive and responsive to environment state changes as it implicitly encourages exploration and thus avoids getting trapped in local optima.

Both algorithms greatly benefit from storing experience of previous steps in the form of $[s,a,r,s{^{\prime}}]$-tuples and sampling from this data during training. This technique called experience replay allows for the reuse of collected data and more flexible data acquisition involving, e.g., multiple simulation workers and combining data of multiple training sessions.

Like many DRL algorithms, these two have difficulties to converge sufficiently when they only receive sparse rewards. As they start out with completely random actions it might be, in the case of tower stacking for example, extremely unlikely that the agent just randomly happens to stack one block on a previous one and thus never receives a positive reward. To enrich this scarce reward landscape, it is common practice to apply reward shaping, in which smaller rewards are granted for actions that, although not entirely satisfying, are still somewhat advantageous. Using TD3 and SAC, different agents were trained within the described framework in two construction-related case studies. The detailed task description, reward-shaping approaches, learning results and robotic execution are described hereafter.

## Case studies

The following two case studies are intended to show the functioning of the distributed training and fabrication setup described in Sect. 2 and further detail fabrication-specific applications. They should be understood as reduced-size demonstrations that, although not quite matching the physical size of actual architectural fabrication, make use of and serve a mode of operation that is very typical in CDRF applications (especially in case study two).

### Case study A: block stacking

#### Setup

**Fig. 2:**
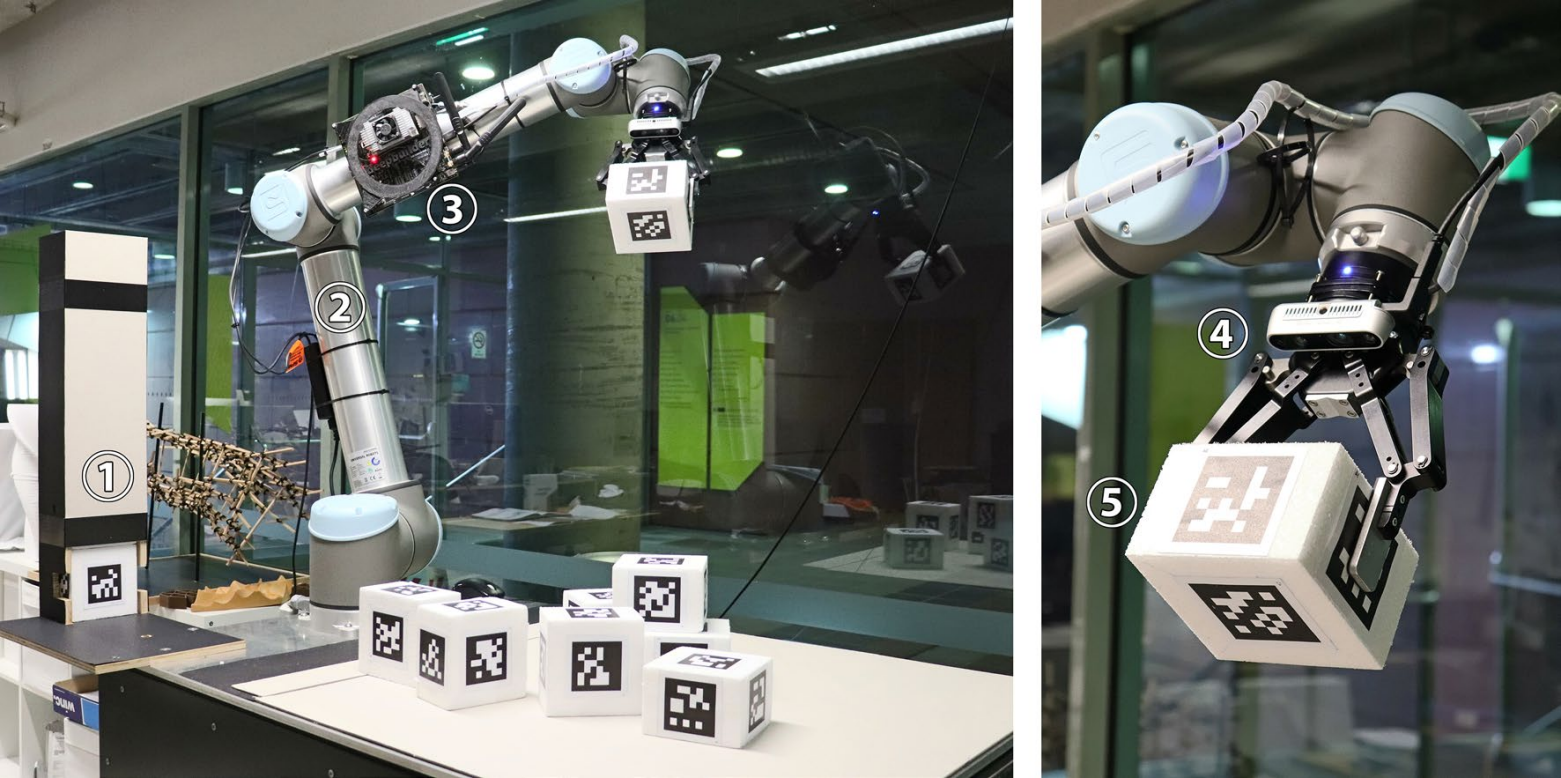
Block building arena: (1) block source; (2) UR10; (3) sensor processing unit Nvidia Jetson TX1; (4) Robotiq 2F-140 two finger gripper and Intel Realsense D435 camera; (5) building block with localization markers attached

In the first case study, the robotic agent consists of a UR10 six-axis robot equipped with hardware shown in Fig. 2. Its task is to stack boxes of 12 × 12 × 8 cm into a tower-like structure. The robot is mounted to a table which limits its reach to the area above its own root plane. It is furthermore confined by a set of virtual safety planes to its left, right, front, back and top. In addition to the table and the safety planes, the block source (Fig. 2: 1) acts as another static collision object of the environment. A sensor-processing unit (3) was used to pre-process image and depth data and wirelessly transmit the results. The boxes consisted of Styrofoam which was manually softened to reduce bouncing. Later cardboard boxes with similar weight, friction and restitution were used. To reduce the reality gap between simulation and execution, the parameters of Flex were carefully adjusted to closely resemble the physical behavior of the actual boxes through visual comparison, trial and error.

#### Task description

By default, the robot rests in a neutral, collision-free home position from which it can reach the block source to pick up a new block. The motion of picking up a new block which starts and ends in the home position is pre-defined and not subject to learning. Actions that are generated by the agent represent a single robot posture consisting of its 6D joint configuration that we call action pose.^7^ Holding a block in its gripper, the robot moves from the home position to the action pose, drops the block by opening the gripper, and returns home. The agent’s task is to find a succession of reachable action poses, from which it drops consecutive blocks in such a way that they form a tower-like structure. At most, the agent can drop 20 blocks into the environment to complete one building attempt or training episode. To plan the motion from the home position towards an action pose, we employ the BiTRRT path planner in MoveIt. Action poses are initially chosen at random from the range [− 180.0°, 180.0] for axes three, four, five and six; axes one and two are restricted to half that range to avoid the most obvious collisions with the floor and virtual safety planes.

The state is represented by 144 (12 × 12) normalized height measurements resulting from the absence or presence of blocks at certain fixed grid points in a quadric section on the table—the play field.

#### Training protocol

Each training epoch begins with resetting the agent’s training environment. This entails:

- clearing any episode-related data and resetting the simulation
- requesting the remote CAD instance manager to start a new CAD simulation instance and return its unique process handle, so this instance can be addressed in the future. If a process handle is already stored from a previous epoch and the respective CAD instance is running, this step is skipped
- ensuring that an active connection to ROS is available^8^ and setting the robot’s pose in ROS to the home position

Once the system is reset and ready the state is processed through the policy network to get an action. The action is passed to the path planner to verify its reachability with the robot. If the action pose cannot be reached the action is discarded, a negative reward is given, and the next action is taken. If path planning is successful, the action is passed to the affiliated CAD instance where the dropping of a block from the action pose is simulated. As soon as the simulation came to a rest, the newly measured state of height field values along with additional info (e.g., exact position of the tower tip or distance from effector to tower tip) is returned to the trainer. This additional info, along with the state is used for reward shaping. Reward and state are returned to the agent which then decides for the next action. Noise is applied to states to avoid overfitting (especially in the early default states where no blocks are present) and prepare the policy for inaccurate measurements during execution later on. The $[s,a,r,s{^{\prime}}]$ tuple is stored in an experience replay buffer. A tower building attempt ends, when the maximum allowed number of actions is reached.^9^ DNN parameter updates are applied between plays at regular intervals.^10^

A typical action cycle would take around 0.1 s for path planning, with an additional 2–3 s for block drop simulation (or around 20 s for real-world robotic execution). Training was performed using the pre-collected data of 1000 random plays as initialization data and 2000 to 3000 additional plays for training. In our framework, a training session of 1000 plays would typically take between 7 and 12 h depending on the number of collisions.

#### Reward shaping

The fundamental measure of success for the agent’s actions is a growth in absolute tower height. In addition to this rarely occurring event, various intermediate reward-shaping measures were taken. The total reward consisted of partial positive rewards (*r*) and negative rewards (penalties (*p*)):

$$r= \left\{\begin{array}{l}{p}_{\mathrm{col}}\quad\text{ if }{\quad}\text{collision}\\ {r}_{\mathrm{prox}}+ {p}_{\mathrm{collapse}}+{r}_{\mathrm{ctrl}}+ {p}_{\mathrm{stuck}}+{r}_{\mathrm{growth}} \quad \text{if }\quad\text{ no collision}\end{array}.\right.$$

*Collision penalty *${p}_{\mathrm{col}}$*:* Self collisions and collisions with the environment caused a severe penalty ${p}_{\mathrm{col}}=-0.25$. In this case, no further reward features were considered and the action was terminated. Otherwise, all other reward-shaping features were summed up as described.

*Tower tip proximity reward* ${r}_{\mathrm{prox}}$: The agent was encouraged to react to the environment by dropping new blocks close to the currently existing tower tip. A short distance ${d}_{c}$ between the highest block in the scene and the TCP position was rewarded. It was measured, normalized to the range ${d}_{c \mathrm{min}}=0.12 m$ and ${d}_{c \mathrm{max}}=0.8 \mathrm{m}$, and further scaled to a maximum value of 0.15:

$${r}_{\mathrm{prox}}=0.15*\frac{\mathrm{min}\left\{ \mathrm{max}\left\{ {d}_{c}, { d}_{c \mathrm{min}}\right\}, { d}_{c \mathrm{max}}\right\} - {d}_{c \mathrm{min}}}{{d}_{c max}- {d}_{c min}}$$

*Tower collapse penalty* ${p}_{\mathrm{collapse}}$: Another penalty ${p}_{\mathrm{collapse}}=-0.05$ was applied, whenever the tower significantly shrank in height after an action. This encouraged the agent to not crash into the tower and keep a certain distance. However, as collapses were often delayed and difficult to predict, its actual effect on learning is debatable.

*Controlled action reward* ${r}_{\mathrm{ctrl}}$: Ideally, the agent should perform controlled actions in which the block, after being dropped, came to rest in a pose that was close to the gripper and of similar orientation. To do so, the Cartesian distance ${d}_{c}$ and quaternion distance ${d}_{q}$ between the poses of TCP and the block (the latter indicating similar orientation) were measured,^11^ normalized to appropriate ranges (${d}_{c \mathrm{min}}=$ 0.12 m, ${d}_{c \mathrm{max}}=$ 0.4 m, ${d}_{q \mathrm{min}}=0.3$ and ${d}_{q \mathrm{max}}=0.8$) and scaled to the range [0, 0.15]. Their average formed ${r}_{\mathrm{ctrl}}$:

$${r}_{\mathrm{ctrl}}= \frac{{\widehat{d}}_{c}+ {\widehat{d}}_{q}}{2}$$

$${\widehat{d}}_{c}=0.15* \frac{\mathrm{min}\left\{ \mathrm{max}\left\{ {d}_{c}, { d}_{c \mathrm{min}}\right\}, { d}_{c \mathrm{max}}\right\} - {d}_{c \mathrm{min}}}{{d}_{c max}- {d}_{c min}}$$

$$ {\widehat{d}}_{q}=0.15* \frac{\mathrm{min}\left\{\mathrm{max}\left\{ {d}_{q}, {d}_{q \mathrm{min}}\right\}, { d}_{q \mathrm{max}}\right\} - {d}_{q \mathrm{min}}}{{d}_{q \mathrm{max}} - {d}_{q \mathrm{min}}}$$

*Block stuck penalty* ${p}_{stuck}$: Accounting for block-robot collisions, a negative reward of ${p}_{\mathrm{stuck}}=-0.05$ was given when the block got stuck in the gripper due to unfavorable effector pose. This encouraged the agent to drop blocks while the gripper was pointing downward.

*Tower growth reward* ${r}_{growth}$: Dropping one block on another one causing the tower to grow resulted in a high additional reward of ${r}_{\mathrm{growth}}=0.7$.

The agent started with random actions. These reward features were laid out to successively encourage it to (a) restrict its actions to the working area (by avoiding ${p}_{col}$); (b) reach for the tip of the existing tower without crashing into it (by increasing ${r}_{\mathrm{prox}}$ and avoiding ${p}_{\mathrm{collapse}}$); (c) exert controlled actions with the gripper facing down (by avoiding ${p}_{\mathrm{stuck}}$ and increasing ${r}_{\mathrm{ctrl}}$), and (d) finally place the new block on top of the existing structure (increasing ${r}_{\mathrm{growth}}$). From this scheme resulted a reward range from − 0.25 for a collision to 1.0 for a perfect action of collecting all rewards and receiving no penalty.

#### Learning results

*Twin delayed deep deterministic policy gradient*

**Fig. 3:**
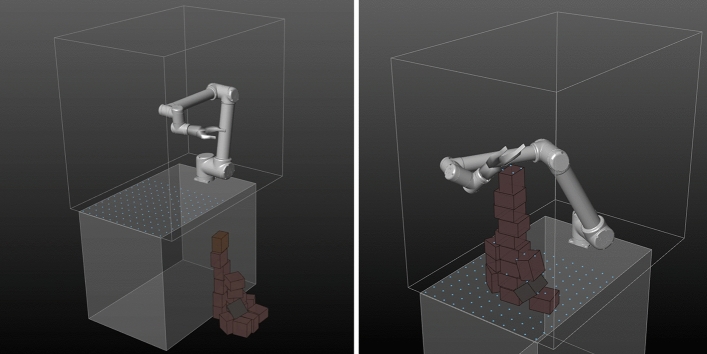
TD3: Agent exploits either singular reward-shaping features such as controlled-action-reward (left) or simulation inaccuracies (right)

**Fig. 4:**
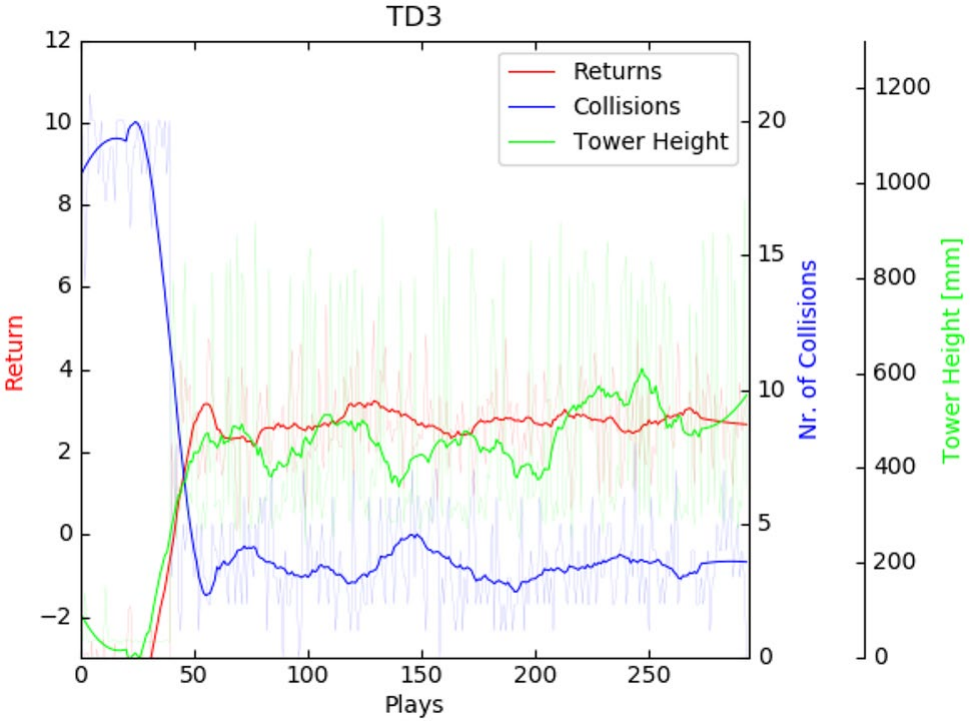
TD3: as collision occurrences disappear the returns and tower heights increase but plateau

Even after thorough hyper-parameter tuning, the TD3-trained policy would quickly converge to a local optimum from which it did not recover. Using a very simple technique of just repeating the exact same suboptimal pose over and over again, the agent is able to effectively avoid collisions and quickly accumulate a lot of controlled-action-reward for small divergence of block orientation (Fig. 3 left, note that no tower growth reward is granted as blocks do not exceed the table in height). In other instances, it successfully builds a tower of around ten blocks by repeating a different pose (Fig. 3 right). This behavior, however, is only successful in simulation as it exploits inevitable inaccuracies concerning Flex’s friction and restitution properties.^12^ Furthermore, achievable tower height is limited due to the robot’s static pose and inadaptability. As soon as the tower reaches the gripper, this strategy fails. This is well reflected in the learning curve’s plateau shape (Fig. 4).

*Soft actor critic*

**Fig. 5:**
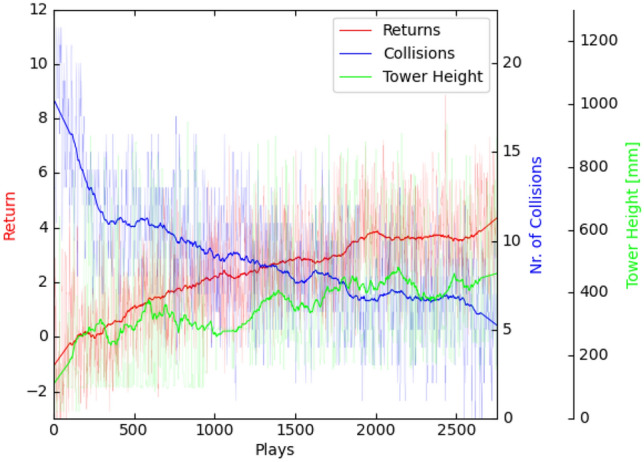
Learning results for SAC in block stacking—learning is generally slower but shows a longer period of improvement

**Fig. 6:**
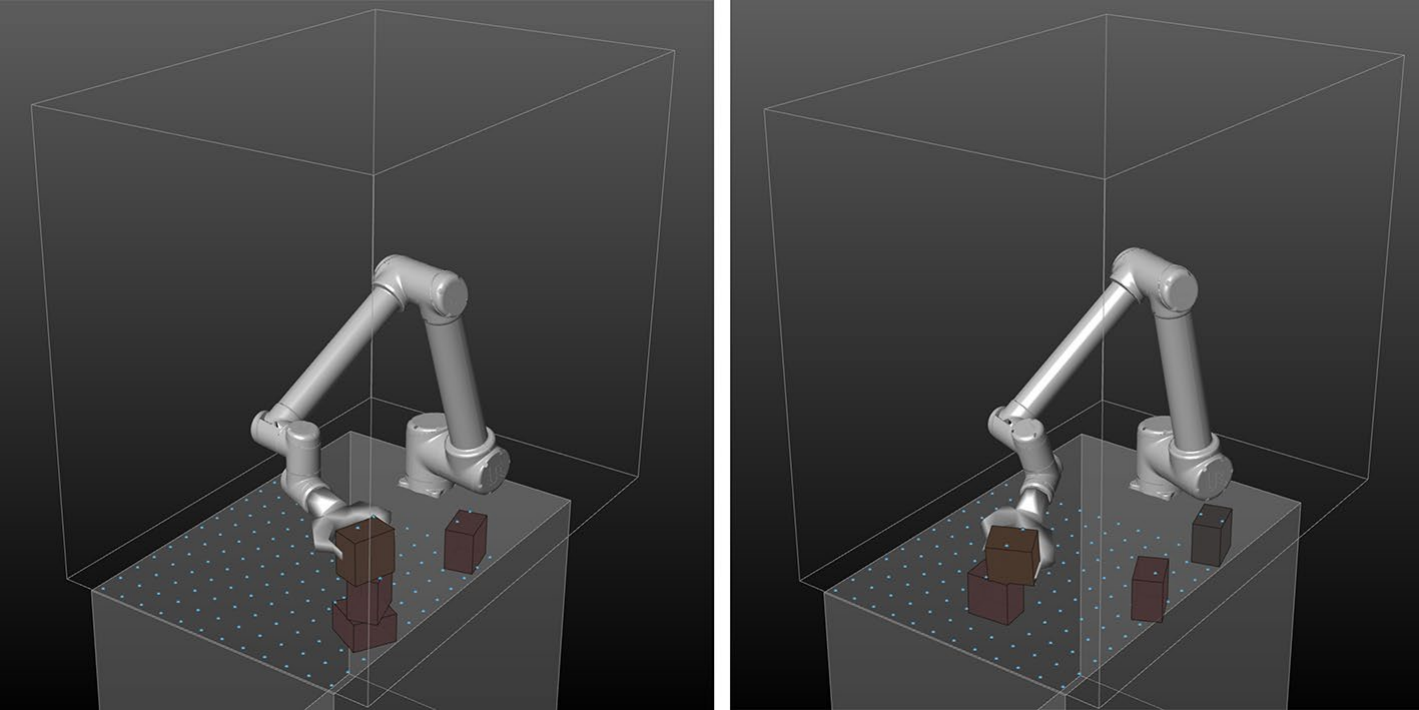
After approx. 600 plays, the agent occasionally performs the best possible action

In contrast to TD3, the agent training with SAC does not quickly converge to local optima and, due to its stochastic policy, generally exhibits a more diverse set of actions (Fig. 5). After around 600 plays, it starts to occasionally perform the best possible action (Fig. 6) where it places a new block on an existing one in a controlled fashion and collects the highest possible reward. Using this technique, it was able to build small towers of up to three blocks. These episodes however remain scarce. With longer training, the agent falls back into somewhat repetitive behavior. However, in contrast to TD3, it reliably choses a better pose from the start (gripper pointing downward, lower position, blocks being positioned not diagonally but with one face parallel to the floor) and reaches for the same points in space from different angles, suggesting that it gradually incorporates some understanding of its forward kinematics.

#### Robotic execution

Both policies were executed on the robotic setup shown in Fig. 2.

**Fig. 7:**
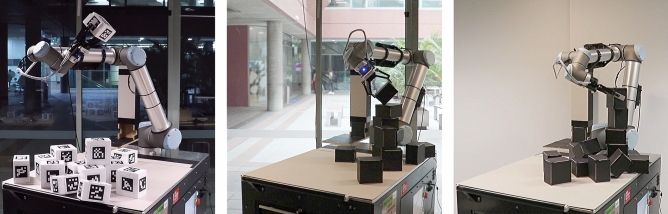
(Left to right) TD3 policy results in unordered block pile; SAC (towers 1 and 2) finds better starting positions and reaches for similar points from different angles, actions are more controlled in SAC

To sense the required 12 × 12 height field, we used the effector-mounted camera’s 1080 × 720 depth field and measured its distance to the blocks/table at specific pixel indices as soon as the robot returned to the home position after each action. To account for inaccuracies in the kinematic chain, the pixel indices were identified at runtime through image compartmentalization and parallel search for depth cloud points closest to those of the fixed grid points in question (with the z-coordinate being ignored during search). This pre-processing was executed on the sensor-processing unit (Fig. 7).

In addition, the aforementioned tag system AprilTag was used for further information about block orientation. This information, however, did not feed into the learning algorithm and was purely used for visual cross-validation.

As mentioned before, the TD3-trained policy fails on the robot as blocks just bounced off uncontrolled after being repeatedly released from the same suboptimal elevated position. The SAC trained policy, however, managed to robustly build towers of four to six blocks in height. Although this success is somewhat aided through slightly heavier building blocks made of cardboard, SAC’s favorable policy is clearly visible. Block building results shown in video two^13^ clearly show SAC’s favorable policy.

#### Conclusion

Case study A was intended to prove the general feasibility of the presented distributed learning framework. The setup of the training environment was made easy using the CAD framework and visual programming interface. Using CAD instance handles, it was possible to run up to four training sessions in parallel on a single simulation machine.^14^ Although the innovative value in robotic learning in this case study is relatively low (autonomous block stacking has been demonstrated before as discussed earlier), it showed that in principle robotic DRL is possible in computational design environments and yields results similar to those of existing studies. This opens up tremendous potentials of using generative design tools as learning environments as was done in case study B.

### Case study B: sensor-adaptive 3D printing

#### Setup

**Fig. 8:**
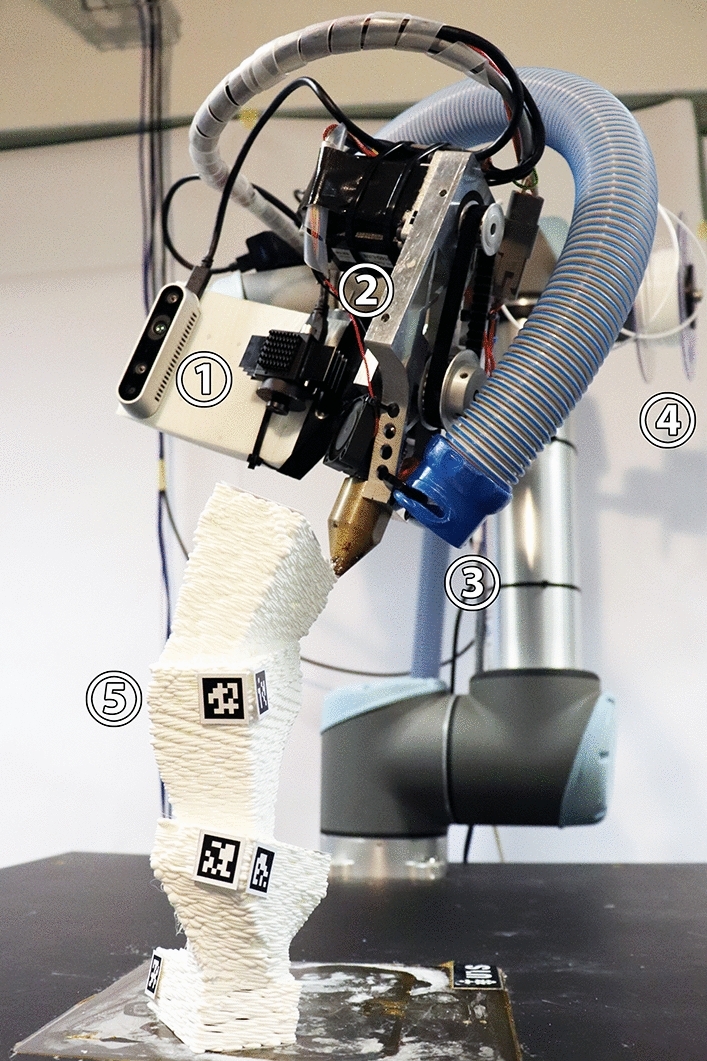
Hardware setup for sensor-adaptive 3D printing: (1) sensor board carrying Intel Realsense D435 and Blackfly BFS-U3-16S2C-CS cameras; (2) custom-made filament extruder with thermoplastic nozzle; (3) vent; (4) filament source; (5) marked print object

**Fig. 9:**
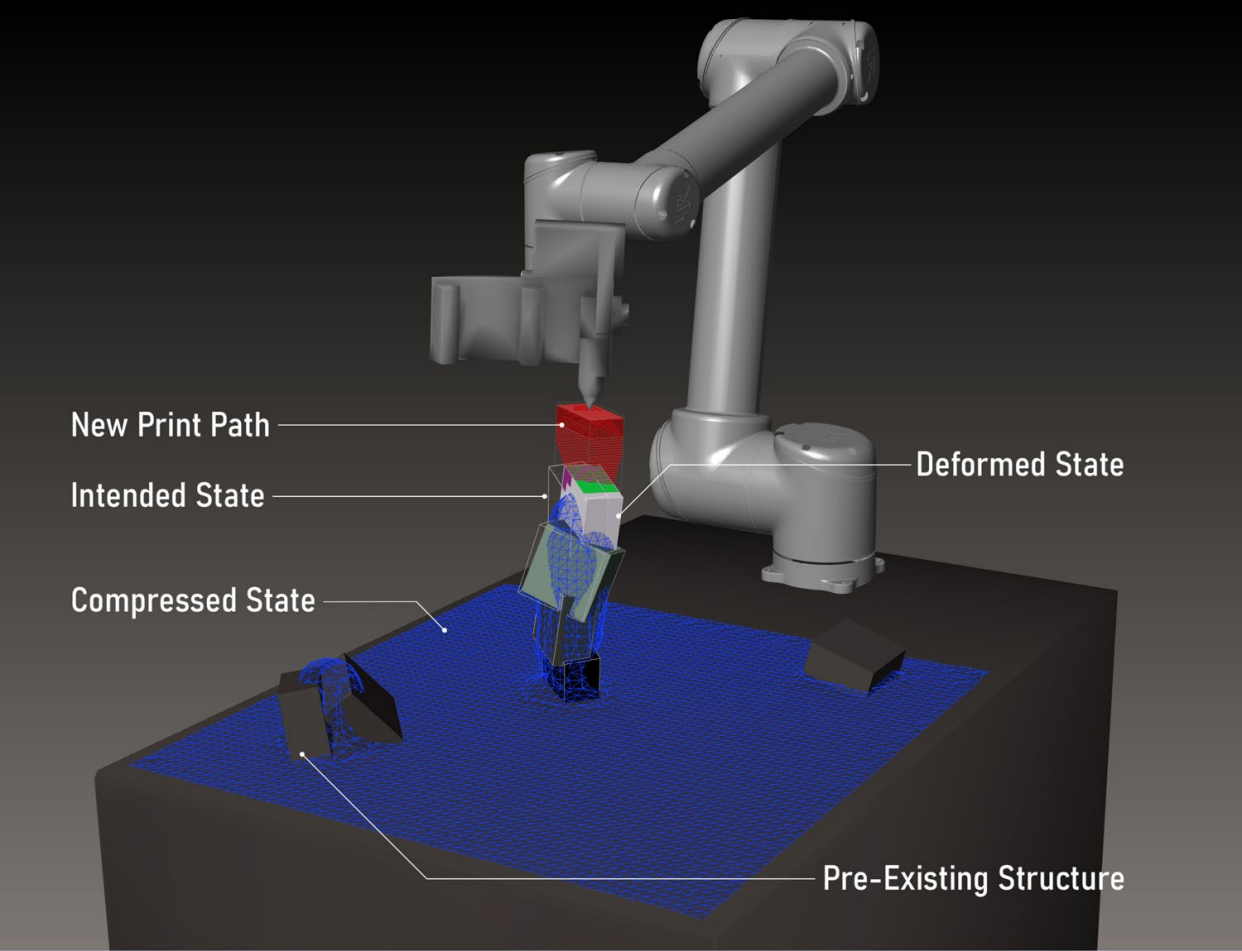
Virtual print environment, the agent action is represented in the robot pose and the rotation angle of new block volume around TCP. Contact surfaces of the state volumes with the new block are shown in pink (non-supportive contact) and magenta (supportive contact area)

In this case study, the same robot arm was equipped with a custom-made 3D printing nozzle capable of extruding different, potentially soft, thermoplastic materials (Figs. 8, 9). The agent’s task was to consecutively add new volumetric segments onto an existing structure through 3D printing. To do so it could sense the existing structure through a tag-based geometry reconstruction system using a high framerate RGB camera. Through this sensory setup, it was able to adapt to unseen starting configurations and react to potential deformations in the structure. This task layout was designed to more closely emulate the requirements of a real-world CDRF problem: a spatially tightly confined robot working with an amorphous material subject to deformation under self-weight in an additive fashion; its controls are differentiated between large global movements to travel between positions and narrow movements to execute local manipulation routines, the planning of which requires high geometric accuracy with respect to a CAD model. Contrary to common CDRF works, however, the global shape of our final product is not a priory human-designed but entirely subject to agent learning.

#### Task description

Printing control is compartmentalized into different categories. Instead of learning low level controls for robot axes and the extruder, the agent’s learned actions represent high-order instructions. Low-level controls are subsequently handled through a parametric tool path planner for printing (similar to a slicer) and machine control in ROS. Like in case study A, the robot starts each training rollout in a neutral home position from which it generates actions in the form a 7D poses, with the first six values again representing a singular robot *action pose*. The action pose’s TCP forms a reference frame, in whose origin a block-shaped volumetric segment—a *candidate*—is situated that might get attached to the existing geometry. It is rotated around the TCP’s Z-axis at the origin by the action’s seventh value.^15^ The immediate goal of an action was to generate a candidate that intersects with and is sufficiently supported by the existing structure, so that it could be printed. The long-term goal was to add segments to the scene to form a structure that stretches in height or covers a large floor area.^16^

#### Training/execution protocol

**Fig. 10:**
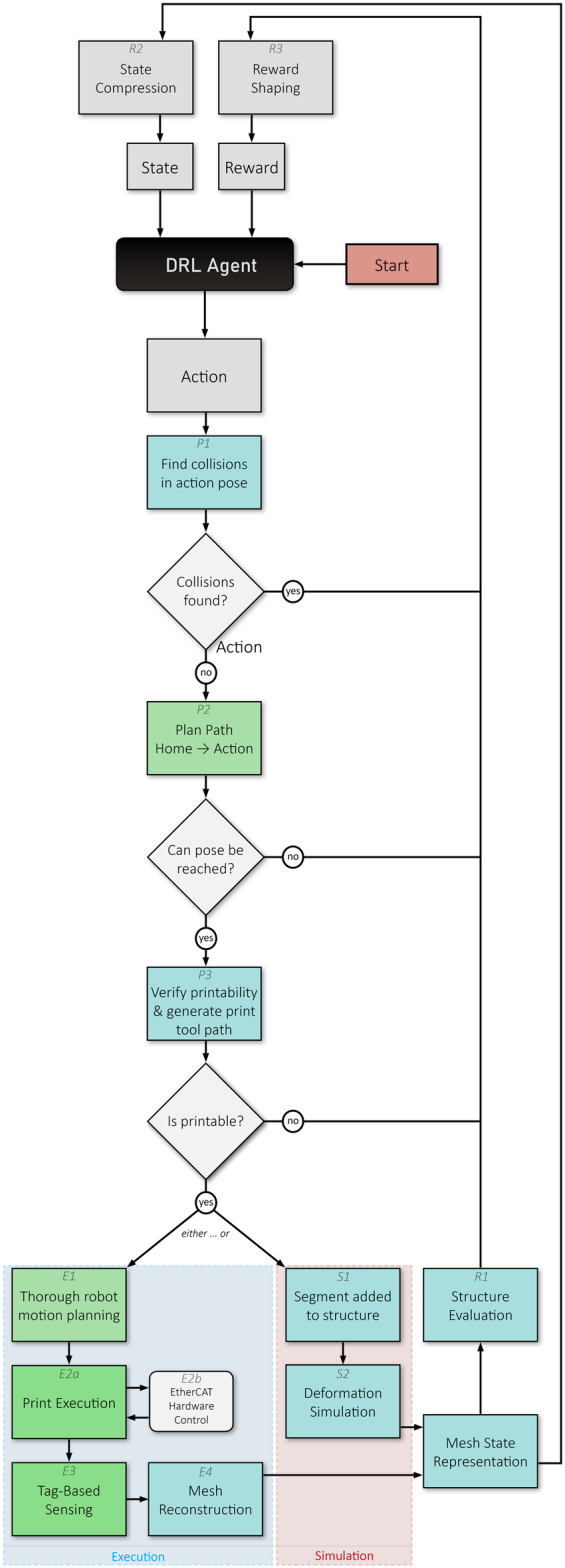
Flow control chart for learning-based sensor-adaptive robotic thermoplastic printing

Figure 10 shows the control flow diagram for this case study. Actions colored in cyan are executed in the CAD environment, the green ones are handled in ROS. These controls are set within the framework shown and described in Sect. 2.1.

*P: pose validation and tool path planning*

**Fig. 11:**
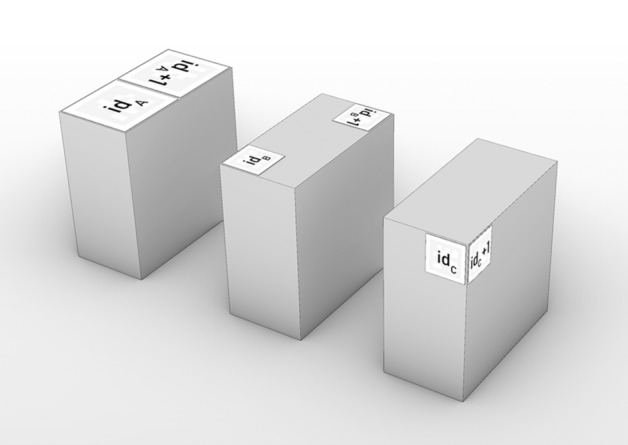
There are three different modes in which a block can be labeled. The available markers are grouped into pairs and categorized in these modes. Markers with an even id in category A, B or C and their successor of id + 1 must be attached according to the picture. With this logic, markers can be attached in different positions on a block to ensure tag discovery from different angles to increase accuracy. An individual block can also be labeled by multiple sets of markers to ensure discovery from different angles

A first simple pose validation (P1 in Fig. 11) by means of mesh intersection quickly filters out unreachable poses. Bad poses are categorized by the objects they cause collisions with (self, table, walls and ceiling, state mesh). Successful poses are further verified by making sure there exists a path from the home position to action pose (P2). Once an action surpassed these steps, it needs to be verified whether its candidate volume can be printed on the existing structure. This is done through a CAD script that evaluates contingent areas of intersection for the existence of surfaces that can support the new candidate (Fig. 9 green, Appendix Fig. 16). It then blends this support area with the desired block shape (hence the “missing corners” in the printed geometry) and slices the resulting volume into layers parallel to the TCP plane. From these layers, the actual print path is generated as a list of Cartesian way points. If this procedure is successful, the action passes and the resulting print tool path, starting and ending in the action pose, is forwarded.^17^

*S: print simulation and training*

Successful candidates are attached to the existing structure. In case of fully rigid material behavior, this step is straight forward: a simple Boolean mesh union of state mesh and candidate. However, it was intended to account for possible deformations in the global structure caused by the added weight of the newly attached segment. Thus, the state geometry is in parallel represented as a position-based dynamics (PBD) particle system using Flex, in which mesh vertices and volumetric particles of an individual segment are grouped together by a shape matching constraint (SMC), making it a pseudo rigid body. When a new segment is added and intersects with existing geometry, its individual SMC is made to incorporate vertices of the existing structure at the area of intersection, effectively connecting the two segments by an elastic link. Thus, the global structure behaves like a semi-soft body.^18^ Whenever a new segment is attached a fixed number of simulation iterations is performed. Damping is added to ensure the system coming to rest.

With this new state mesh, relevant information about the action’s outcome can be drawn, such as height of the structure, covered floor area, relative deformation (as the per-segment displacement from the segment’s initial center plane position both in Cartesian and quaternion space), distance from the TCP to the structure and more (R1). This information is then used for reward shaping (see R3).

*E: robotic execution*

The CAD-generated tool path is used for robotic Cartesian motion planning in MoveIt (E1) and the print procedure is executed with the robot (E2a). During this process the extruder motor as well as heat and fan control, which are controlled via EtherCAT fieldbus, are monitored and adjusted through a ROS node communicating with TwinCAT-Http-Server (E2b). As is often the case with 3D printing, this extrusion is quite slow. Printing a single segment typically took between one and three hours, depending on its actual geometry, layer thickness and movement speed.

Once printing has finished, a human operator attaches April tags onto the printed object following a few simple rules (Fig. 11) so that the geometry can be scanned with the effector-attached RGB camera.^19^ To increase precision and reduce blind spots, multiple tag measurements are taken while the robot moves along a parametrically defined simple discovery path (a circular motion above the last action TCP position with the camera facing inward). At the end of this procedure, tag measurements concerning one and the same block are merged via Cartesian and quaternion averaging with larger tags of mode A being weighed higher. The averaged block positions are related to the measurements of fixed-position reference markers on the arena’s corners and merged into an updated mesh-based state representation (Fig. 10: E4). Through this sensing method, a high accuracy with a maximum observed deviation of two millimeters was reached.

Through this sensing method, a high accuracy with a maximum observed deviation of 2 mm was reached. This was especially useful as the measured geometry served as a basis for future print tool path generation (P3).

*State compression R2*

In case study A, the state could be sufficiently represented by a 12 × 12 grid of height values. Case study B, however, investigates a more complex scenario in which the state is constituted by actual volumetric geometry. A consistent and expressive method of geometric encoding was, therefore, crucial. With the intention for general applicability in mind, hand crafted and task-specific feature extraction, e.g., through extraction and parametrization of geometric primitives was not suitable. Multiple studies demonstrated the potential of deep learning with signed distance field (SDF) data as an efficient means of volumetric shape compression, interpolation and completion (Angela Dai et al. 2016; Park et al. 2019). Thus, the use of a convolutional autoencoder (CAE) to encode the state by means of SDF data compression stood to reason.

Another viable approach is the extension of principle component analysis (PCA) to higher dimensional data sets by means of tensor rank decomposition. This method has been discussed and demonstrated in other realms of data compression (Chen and Shapiro 2009; van Belzen and Weiland 2012) and visual 3D data (Ballester-Ripoll et al. 2019) and has the advantages of not requiring lengthy training computation and not being prone to faulty parameter tuning. Furthermore, as the number of rank-1 tensors per dimension utilized for decomposition can be chosen at runtime, the degree of compression (and loss) can be tuned without the need for retraining. In such a scenario, the state could consist of the flattened decomposition tensors. As an application of tensor decomposition to SDF data to our knowledge has not been presented yet, a comparison with the aforementioned CAE approach was undertaken. Specifically the CANDECOMP/PARAFAC decomposition (Carroll and Chang 1970; Richard and Harshman 1970) was performed on 64^3^ SDF data.

The utilized CAE had the following structure: a 64^3^ tensor containing the SDF data was processed through three consecutive convolutional layers (kernel size 4, stride 2, interposed batch normalization, ReLU activation) of edge size 64, 32 and 16. After flattening the tensor, the data was processed through three fully connected layers of size 1296, 432 and finally 144 (or 256) units—the bottleneck—whose outcome is presented to the agent as the state. The decoding pipeline is of the same, but mirrored structure. Two such networks were trained on around 100k mesh samples^20^ using an SGD optimizer.

**Fig. 12:**
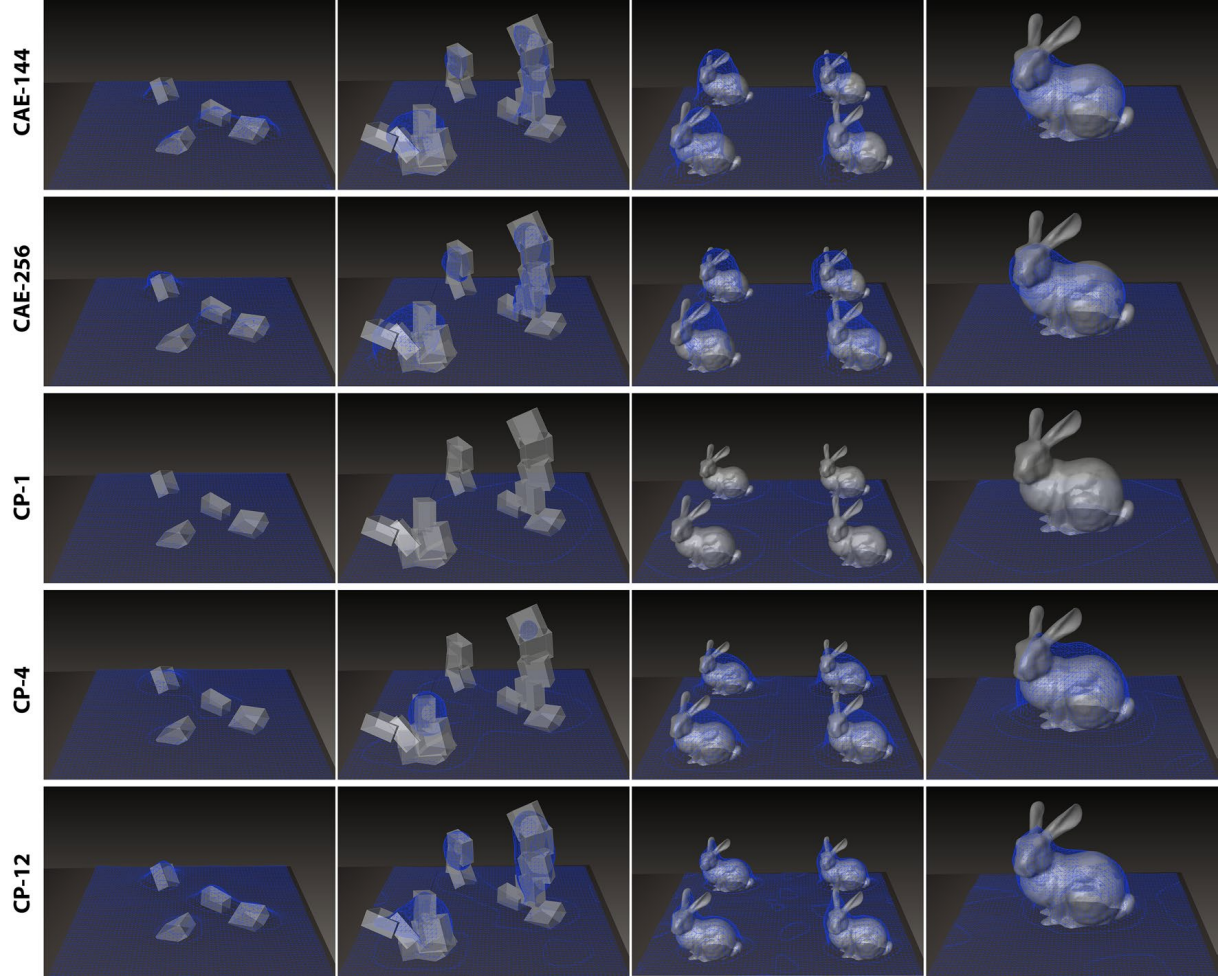
Comparison between 64^3^ SDF compression methods for state representation: convolutional autoencoders (CAE) of different bottleneck sizes are compared to nD-PCA via CANDECOMP/PARAFAC (CP) tensor rank decomposition using different numbers of rank-1 tensors per dimension (1, 4, and 12): **a** CAE-144: bottleneck size: 144, compression ratio: 99.945%; **b** CAE-256: bottleneck size: 256, comp. ratio: 99.902%; **c** CP1: bottleneck size: 192, comp. ratio: 99.927%; **d** CP-4: bottleneck size: 768, comp. ratio: 99.707%; **e** CP-12: bottleneck size: 2304, comp. ratio: 99.121%. CAE exhibits much better reconstruction results (blue) from the input meshes (white) at higher compression rates. To achieve the same reconstruction quality CP requires wider bottlenecks. Furthermore, no considerable performance difference can be observed between CAE-256 and CAE-144, rendering the latter as the superior compression method

A comparison of reconstruction results is shown in Fig. 12. With an average MSE of consistently under 5*10^–5^ on unseen data and visually more satisfactory reconstruction at much higher compression ratios, the CAE of bottleneck size 144 was chosen for state compression.

*Reward shaping R3*

**Fig. 13:**
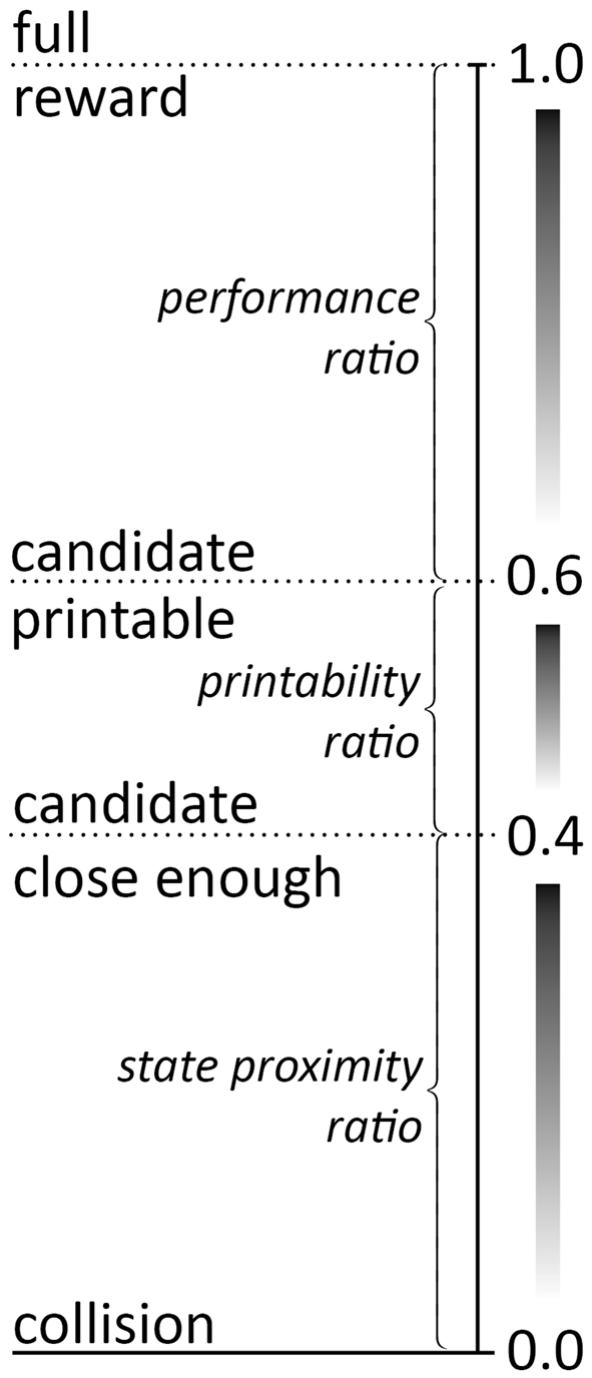
Reward scale for case study B

The agent’s general goal is to build a structure that grows in height or in covered floor area (preferably both) while deforming as little as possible. Reward shaping was designed to gradually guide the agent from (0) random actions with many (self) collisions towards (1) less collisions to (2) reachable action poses, (3) printable segment candidates to (4) increased structural performance of these candidates in the mentioned criteria. An intuition of the relationship between these criteria is summarized in Fig. 13. No reward is granted if a collision occurs. If the agent avoided a collision, it could earn a small reward of up to 0.4 when its TCP and thus the candidate segment are close to the existing state mesh. This state proximity ratio is the sum of (a) the normalized inverse Cartesian distance between the TCP and the closest point on the existing state mesh and (b) the normalized deviation of the TCP’s orientation from the ideal downward-pointing pose, i.e., the angle between TCP z-axis and global z-axis. The latter takes the issue with tilted printing layers into consideration: Nozzle orientations and thus printing layers diverging more than 45° from the horizontal plane are considered unprintable and thus penalized. The closer the TCP is to the existing structure and the closer its pose is to facing downward, the more likely it is that the action produces a printable candidate segment, and thus the higher the state proximity reward. With actions getting closer to the existing structure, a printability ratio signals if and how well a candidate segment can be printed. It is zero if (a) no candidate-state mesh intersection is found or it is too small, (b) the segment would be attached laterally instead of on top of the structure or printing is blocked for other reasons. If none of these negative circumstances occurs, the printability ratio is the normalized inverse intersection volume between state and candidate. It is a simplified, faster substitute for generating an actual print path with the aforementioned CAD script (which is costly). This encourages the agent to choose actions that would yield a valid and preferably long print tool path resulting in larger segment volumes. If the printability ratio is larger than zero, and thus a candidate segment is printable the agent can earn an additional structural performance reward: the minimum of either (a) the height increase normalized by the maximum possible per-action height increase: a full segment height; or (b) covered area increase normalized by the maximum possible per-action area increase: the area of a segments front face. This performance ratio is scaled by the average per-segment displacement through deformation normalized by a particularly chosen maximum deformation (2 cm average segment displacement was considered the worst), to discourage large deformations in the printed structure.

#### Learning results

**Fig. 14:**
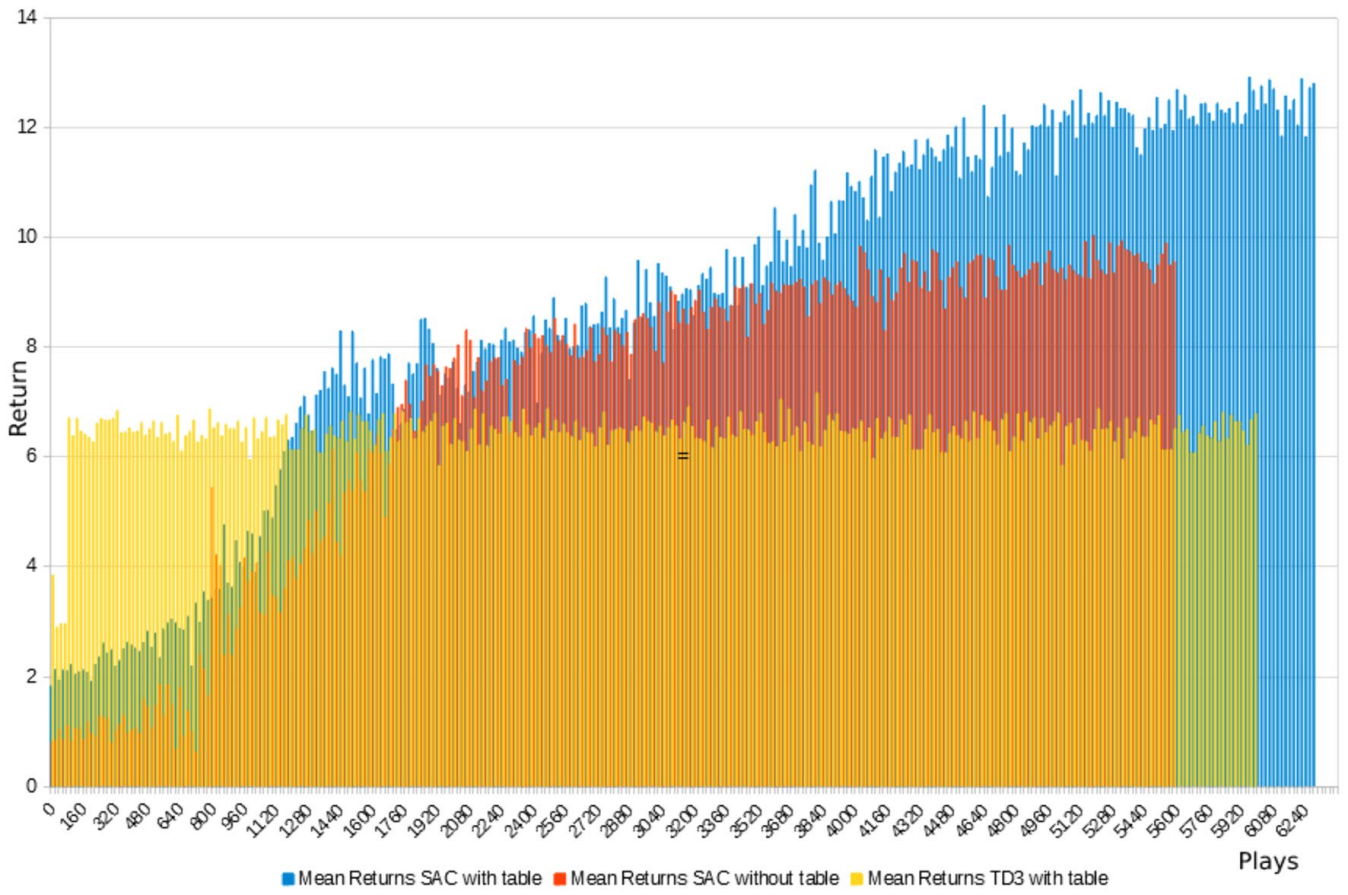
Comparative learning success between TD3 and SAC with and without the ability to initialize structures

**Fig. 15:**
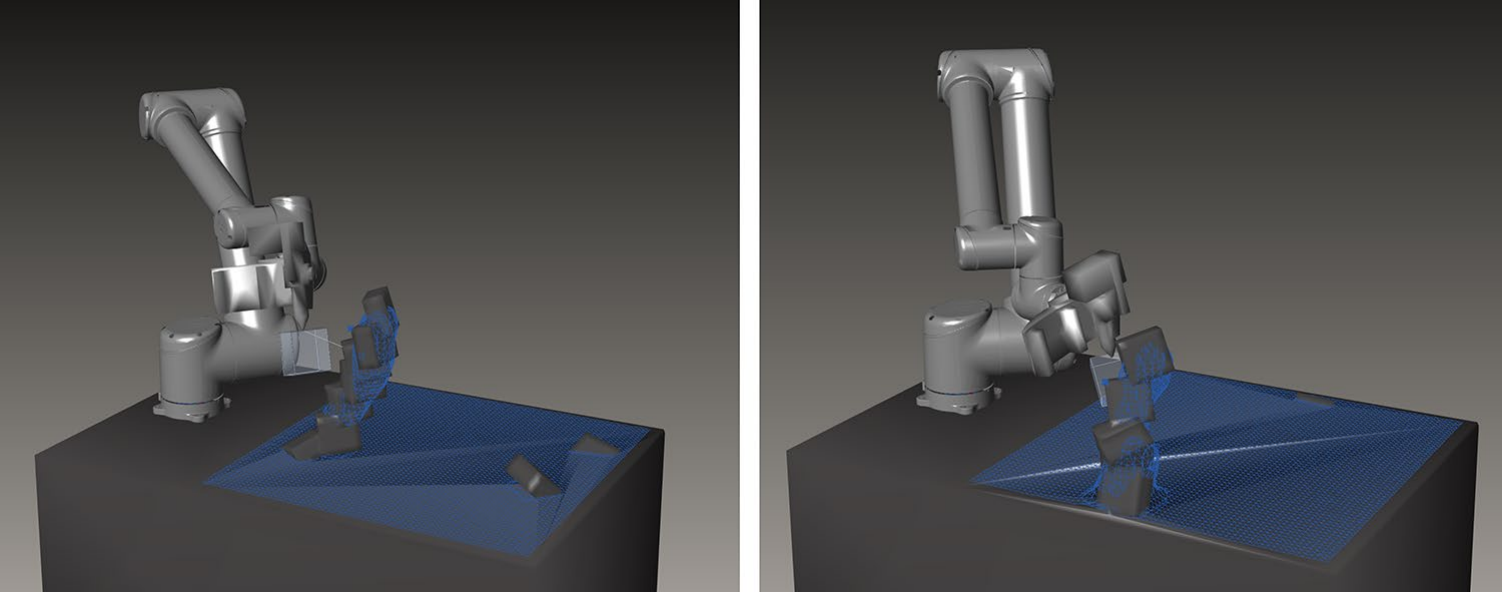
Autonomously planned towers using SAC

A training episode consists of 30 actions after which the printed structure is reset. Training incorporated the data of 100 k pre-collected action samples. Actions range from − 45° to 45° for axes one, two and three and from − 90° to 90° for the others. In case study A, a certain indifference of the agent toward states was observed. This was partly because it always started out with an empty table and could start building a tower wherever it wanted, leading to somewhat repetitive behavior. To counteract this tendency, episodes in case study B were initialized with randomly placed existing blocks enriching the state information from the very start. In this scenario, it is significant, whether the table mesh is treated as part of the state or not. If so, the agent can start building structures directly on the floor without the precept to incorporate information about present blocks. If not, the agent is not allowed to seed new structures and needs to find an existing block to build on. Figure 14 shows the comparative learning success of SAC and TD3 with and without permission to initialize structures. Naturally, the mean returns are higher from the start when this permission is granted as state proximity ratios are also measured from the table mesh and random successful prints occur more often. Agents trained with TD3 very quickly retreat to a safe, collision-free pose and collect proximity rewards if initial building blocks happen to be nearby, but do not leave this pose or explore the environment. Even in the comparatively easier task where building on the table is allowed, no deliberate building is observed. On the contrary, an agent trained with SAC reliably prints towers after around 4500 episodes. It initializes a tower at a random, yet mostly on the left half of the table, and adds new segments on the top of the tower tip by moving its nozzle up after a successful print. Figure 15 and video three^21^ show this learning success in more detail and with varying material rigidity. If the permission to structure initialization is not granted, the SAC agent performs considerably worse (TD3 was not even tested in this more difficult scenario). This suggests that it (a) has difficulties to correctly identify the position of present blocks in the scene and/or (b) successful print events on existing blocks occur too rarely for it to draw conclusions from them. The policy that works, however, produced structures of up to seven blocks. It also seems that these towers tend to grow diagonally (Fig. 15 left). Whether this happens by accident or due to deliberate planning to increase the covered area could not be determined at this point. It must be noted, that the produced actions all represent fabricable building blocks, as the existence of a motion plan toward the action pose and of a valid print tool path are inherent performance criteria in learning. Due to government measures implemented to combat the Covid-19 pandemic in Australia, the trained policy could not be deployed on the physical robot anymore. With all software and hardware components laid out, however, the real-world implementation is straight forward.

## Conclusion

### Achievements

The presented workflow combines quasi-standard tools of robotics, DRL and CDRF research into an integrated agent training and control environment for autonomous robotic construction. It is open to arbitrary model-free DRL algorithms via the OpenAI Gym interface and offers simple environment setup through a well-established visual-programming interface. Yet, its language-agnostic microservice layout in principle allows for the use of any backend software as a simulation environment. It furthermore allows the use of CAD scripting routines for sophisticated geometric representations and to complement agent actions. It also enables real-time industrial machine control common in industrial fabrication setups. Through CAD instance management and error handling, uninterrupted training sessions of multiple days to weeks can be performed, although stability decreases with the number of parallel training sessions.

The framework’s operation was demonstrated in two construction-related case studies, serving as stand-ins for large-scale fabrication scenarios, in which commonly arising issues such as sensor-adaptive automation, structural as well as tooling-related planning, geometric state representation and real-time control are addressed. The case studies presented ways of formulating DRL learning as a succession of independent constructive actions contributing to a global structure. The learning performance of SAC and TD3 within these exemplary tasks could successfully be compared. SAC has proven to be the more effective training method for our use cases which conforms to the scientific consensus within DRL research.

### Shortcomings

In terms of simulation speed, the presented training framework does not compare favorably to highly optimized simulation software like MuJoCo. Although a considerable performance increase could be observed when thorough collision checking and path validation in MoveIt are skipped in favor of simple mesh intersection in CAD, its distributed nature and processing through multiple network layers as well as relatively slow visual scripting computation represent bottlenecks. Although geometric CAD scripting was found to be a promising means to simulate construction, the development of tool path planning subroutines that account for every possible geometric configuration of state and action is difficult. Thus, our print path generation script would not produce feasible outputs in about 5% of the cases. Furthermore, structural performance analysis by means of PBD as done in our case studies does not represent a sufficient means of stress evaluation in construction and was chosen mainly for simulation speed. Furthermore, its actual impact on the learning procedure in case study two can be questioned as the agent did not seem to make such high level considerations.

In a more general scope, the structures that were produced with our methods do not represent useful architectural objects.

### Future work

As this study was focused on using comparatively simple and easy-to-adapt learning algorithms, model-based RL approaches were not considered. However, construction scenarios in which system dynamics are well known, e.g., construction of fully rigid objects or FEA-based stress and deformation analysis, model-based learning could tremendously increase sample efficiency. Within the model-free realm more efficient information-extraction methods such as Hindsight Experience Replay (HER) also bear a great potential in reducing training time, given that well defined goal descriptions are provided. In this context, the formulation of structural performance goals could be considered. In terms of performance evaluation, a multitude of aesthetic and structural criteria is thinkable. In this regard, actions can be used to further parametrize geometric modules to produce visually more pleasing outcomes. The successful creation of more complex architectural objects with the presented approach stands or falls with a carefully crafted reward-shaping method. There exists a tradeoff between allowing unforeseeable scenarios to unfold and defining specific geometric aspects of reward shaping to steer agent behavior. Using SAC for training, we found that more action-specific feedback (like rewarding a short effector distance to the current tower tip) leads to more effective learning than higher-level structural goals (like overall tower height or covered floor area). The agent also benefits from a reward shaping that encourages small consecutive improvements over distant high rewards. In our example, this succession was (1) collision avoidance, (2) robot effector pointing downward, (3) getting near the existing structure and finally (4) attaching a new piece.

Big potential in terms of real-world application of our approach lays in the use of larger, high-payload industrial robots. Although readily available at the time, their use was not favored due to the experimental nature of the study and safety concerns. Deployment onto such machinery necessitates serious considerations of appropriate safety measures. The benefits of a real-world application could be less time-consuming tool path generation, a more tightly integrated manufacturing procedure that mediates between and optimizes for structural criteria as well as fabrication constraints, and ultimately a higher degree of construction automation with benefits for efficiency and human health. In this context the presented work can best be understood as a proof of concept to be adapted for even more powerful learning algorithms in the future whose actions are very closely monitored by a human—the ultimate actor-critic.

## Electronic supplementary material

Below is the link to the electronic supplementary material.

Supplementary file1 (MP4 6940 KB)

Supplementary file2 (MP4 25650 KB)

Supplementary file3 (MP4 71487 KB)

## Figures and Tables

**Fig. 16:**
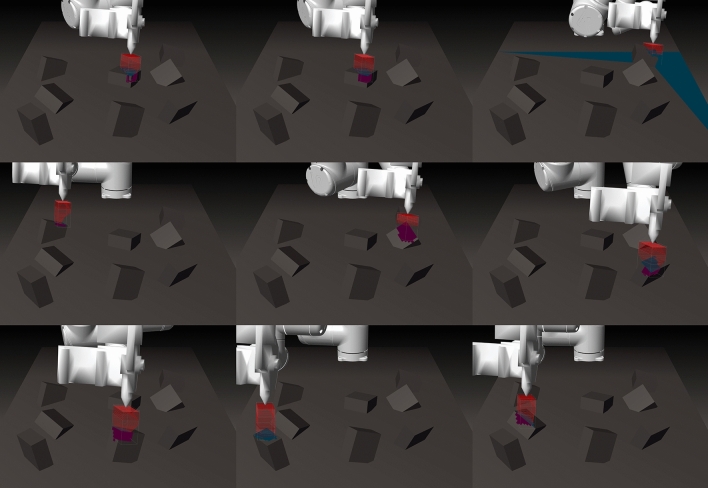
A CAD script indicates whether a print is possible based on different environmental conditions and returns the appropriate tool path. Potential support surfaces are shown in green, non-supportive intersection surfaces are magenta

## Appendix

The learning parameters for DRL algorithms:

**Table:**

Training algorithm	Case study A	Case study B
Both		
Optimizer	Adam	Adam
Reward range	− 0.25 to 1.0	0.0 to 1.0
Hidden layer sizes (all networks)	[256, 256]	[432, 360]
Activation function	ReLu	ReLu
Value net learning rate	0.0003	0.0003
Soft Q learning rate	0.0003	0.0003
Policy learning rate	0.0003	0.0003
TD3		
Discount	0.99	0.99^a^
Batch size	256	256^a^
Network gradient steps per action	0.625	1
Actions between network updates	800	600
SAC		
Discount	0.99	0.9^a^, 0.99^b^
Batch size	256	128^a^, 256^b^
Network gradient steps per action	1	1
Actions between network updates	200	600
Target smoothing coefficient ($\tau$)	0.005	0.05^a^, 0.005^b^

^a^Experiment in which agent was allowed to initialize new structures, ^b^agent was not allowed to initialize new structures

The optimizer parameters for CAE training for state compression:

**Table:**

Optimizer	Stochastic gradient descent
Optimizer momentum	0.7
Learning rate	0.02
Weight decay	0.0

See (Fig. 16).

## Footnotes

1 Electronic supplementary material 1: video 1.

2 Source code available at https://github.com/HeinzBenjamin/deepbuilder.

3 Driver configuration specific to our setup available at https://github.com/HeinzBenjamin/deepbuilder-catkin.

4 CAD applications such as Rhino-GH are often based on the.Net framework and typically not designed for and thus not robust towards inter-process communication. Thus, to avoid crashes special care had to be given to synchronizing incoming traffic with the GH update cycle and thread. This circumstance prohibited more direct communication via ROSBridge and necessitated dedicated instance management capabilities.

5 For this study, the http functions of the Bengesht plugin were used: https://github.com/behrooz-tahanzadeh/Bengesht.

6 Source code available at https://github.com/HeinzBenjamin/TwinCAT-Http-Server.

7 We could have also chosen to represent actions with Cartesian coordinates of the effector frame with its rotation defined as Euler angles or quaternions. With readily available IK solvers, this would have enabled a more intuitive interpretation of actions. However, joint space coordinates were preferred to due to the absence of singularities and their categorical consistency (i.e., no differentiation between position and rotation, like in Cartesian space) which we considered beneficial to learning.

8 Via roslibpy—https://github.com/gramaziokohler/roslibpy.

9 In previous attempts, collisions with the state were made to terminate a play. However, as reset routines are computationally more time consuming and no better training results could be observed, a fixed length trajectory was favored.

10 For learning, the open-source tool RLkit was used and reasonably modified: https://github.com/vitchyr/rlkit.

11 With quaternion, distance ${d}_{q}$ here being the angle of the shortest arc between poses *q*1 and *q*2.

12 Flex only allowed for simulations with slightly too low restitution and too high friction compared to the real world, leading to cubes coming to rest quicker in the simulation. A real box dropped from the high effector positions that TD3 produced would bounce off the table and be lost. For SAC, this effect was less severe, as it produced poses that were lower and caused less bouncing. This aspect did not affect case study 2, as drops influenced by restitution were not part of the simulation.

13 Electronic supplementary material 2: video 2.

14 Intel Core i7 7700 K, GTX1080, 32 GB RAM, with RAM being the limiting factor.

15 A rather simple cuboid candidate shape was chosen, mainly to simplify robotic scanning and tag-based shape reconstruction. However, in principle any geometric form can be used as the candidate geometry. They could also be further parameterized through agent actions, instead of just rotating them around the TCP’s Z-axis.

16 Covered floor area meaning unsupported area. Footprint of the state structure was discarded.

17 Note that in training simulation this is simplified with the concept of a printability ratio, which is explain later on. However, in this step all kinds of fabrication and tooling related criteria could be used for feedback to the agent.

18 The ambition of this soft body simulation was not physical accuracy but simulation speed. However, parameters such as SMC stiffness and damping were tuned to behave like a soft 3D printing thermoplastic like NinjaFlex.

19 This procedure can potentially be sped up using a 3D scanner with millimeter precision. However, such a device was not available at the time.

20 Around 5600 solid meshes were downloaded from Thingi10K and procedurally manifolded through non-uniform scaling, bending and twisting. Gaussian noise of 1.5 mm standard deviation was added to the SDF data drawn from the samples. The network was trained on a GTX1080 for around three weeks.

21 Electronic supplementary material 3: video 3.
